# Predicting the risk of hypertension using machine learning algorithms: A cross sectional study in Ethiopia

**DOI:** 10.1371/journal.pone.0289613

**Published:** 2023-08-24

**Authors:** Md. Merajul Islam, Md. Jahangir Alam, Md Maniruzzaman, N. A. M. Faisal Ahmed, Md Sujan Ali, Md. Jahanur Rahman, Dulal Chandra Roy

**Affiliations:** 1 Department of Statistics, Jatiya Kabi Kazi Nazrul Islam University, Trishal, Mymensingh, Bangladesh; 2 Department of Statistics, University of Rajshahi, Rajshahi, Bangladesh; 3 Mainanalytics GmbH, Sulzbach/Taunus, Germany; 4 Statistics Discipline, Khulna University, Khulna, Bangladesh; 5 Institute of Education and Research, University of Rajshahi, Rajshahi, Bangladesh; 6 Department of Computer Science and Engineering, Jatiya Kabi Kazi Nazrul Islam University, Trishal, Mymensingh, Bangladesh; Lorestan University of Medical Sciences, ISLAMIC REPUBLIC OF IRAN

## Abstract

**Background and objectives:**

Hypertension (HTN), a major global health concern, is a leading cause of cardiovascular disease, premature death and disability, worldwide. It is important to develop an automated system to diagnose HTN at an early stage. Therefore, this study devised a machine learning (ML) system for predicting patients with the risk of developing HTN in Ethiopia.

**Materials and methods:**

The HTN data was taken from Ethiopia, which included 612 respondents with 27 factors. We employed Boruta-based feature selection method to identify the important risk factors of HTN. The four well-known models [logistics regression, artificial neural network, random forest, and extreme gradient boosting (XGB)] were developed to predict HTN patients on the training set using the selected risk factors. The performances of the models were evaluated by accuracy, precision, recall, F1-score, and area under the curve (AUC) on the testing set. Additionally, the SHapley Additive exPlanations (SHAP) method is one of the explainable artificial intelligences (XAI) methods, was used to investigate the associated predictive risk factors of HTN.

**Results:**

The overall prevalence of HTN patients is 21.2%. This study showed that XGB-based model was the most appropriate model for predicting patients with the risk of HTN and achieved the accuracy of 88.81%, precision of 89.62%, recall of 97.04%, F1-score of 93.18%, and AUC of 0. 894. The XBG with SHAP analysis reveal that age, weight, fat, income, body mass index, diabetes mulitas, salt, history of HTN, drinking, and smoking were the associated risk factors of developing HTN.

**Conclusions:**

The proposed framework provides an effective tool for accurately predicting individuals in Ethiopia who are at risk for developing HTN at an early stage and may help with early prevention and individualized treatment.

## Introduction

Hypertension (HTN), defined as the elevated blood pressure beyond its normal ranges, is a major public health concern with its raising prevalence and effect among the adults’ overtime worldwide [1–3]. It is one of the most common serious chronic non-communicable diseases. Hypertensive people are affected by different types of cardiovascular diseases (CVDs), e.g., coronary heart disease, stroke, peripheral arterial disease, aortic disease, myocardial infarction [4–7], which are the leading cause of disability, morbidity and mortality that increase the economic burden of out-of-pocket expenditures (OOPE) [8–10]. As reported by World Health Organization (WHO), worldwide around 9.4 million people were died due to HTN every year [10]. According to Belay et al., [2022], globally the prevalence of HTN was 26% in 2000 and it was projected to reach around 1.56 billion (29.2%) by 2025 [11]. The latest estimation by WHO in 2021 revealed that about one-third (31.1%) of the world’s adult population had HTN (1.39 billion); of whom 2/3 were from in low and middle-income countries (LMICs) [12]. Also, a systematic analysis of population-based studies from 90 countries, including Ethiopia estimated that HTN among adults was more prevalent in LMICs (31.5%) than the high-income countries (28.5%) [13]. Different epidemiological studies in Ethiopia reported that the prevalence of HTN was ranging from 7.7%-41.9% [14]. Moreover, the prevalence of HTN is disproportionately more prevalent and it increases alarmingly in poor resource countries, like Ethiopia [11]. But it might be helpful to mitigate and manage/control the risk of HTN if identification of HTN patients with interpretable risk factors at an early stage. Thus, early detection of HTN patients with identification of interpretable risk factors plays a key role, which could help to get the patients timely prevention and intervention. It is therefore highly essential to detect/diagnosis and identify the interpretable risk factors of HTN at an early stage.

Many convincing research and empirical studies determined several risk factors associated with HTN in LMICs countries, including Ethiopia [15–21]. Nevertheless, existing association studies had several limitations. Most importantly, previous existing studies considered traditional linear models, such as logistic regression (LR), Cox proportional hazard model, for identifying the significantly associated risk factors of HTN [22–24]. Moreover, a real data with high-dimensional non-linear pattern presents a challenge to traditional linear models, and low precision of linear models impedes patients-level use. To overcome those limitations with complex real data, machine learning (ML) might be a right choice, which is being widely used in current public health research fields. ML is a subset of artificial intelligence (AI), in which the algorithms that execute the prediction process collect the necessary information from previous experiences and/or detect patterns in data to accomplish a task, typically a classification or identification [25–28]. It can provide several advantages, including automatic specific process, reliable probabilistic estimation for uncovering hidden patterns or relationships with high accuracy while lowering labor costs and time for large amounts of data that aid in decision-making or inference, and model interpretability [29–31]. There are different types of learning algorithm in ML, among them supervised learning is the most popular and widely applicable. The supervised learning algorithm’s goal is to use the dataset to build a model that can predict the system’s output given new inputs. The major two types of supervised learning algorithm are regression and classification. Example of regression include linear regression and logistic regression [32]. Examples of classification include ensemble methods, decision trees (DT), k-nearest neighbors (kNN), support vector machine (SVM), Naïve Bayes (NB), artificial neural network (ANN), so on [32, 33]. The ensemble method is a machine learning technique that combine multiple models with the same learning algorithm to achieve better predictive performance [34]. Ensemble methods include eXtreme gradient boosting (XGB), adaBoost, histogram-based gradient boosting classification Tree, and random forest (RF) [25]. However, previously, some researcher’s conducted their study to develop multivariable prediction models using several ML and explainable artificial intelligence methods [35–37]. Most of the existing risk prediction models were developed with limited number of risk factors that provided less accuracy for predicting HTN patient [35, 38]. However, DT and ensemble approaches have attracted a great attention in recent years for identifying individuals at risk of HTN, there is no evidence that these algorithms are successfully applied in Ethiopian clinical settings.

To the best of our knowledge, this is the first study that applied and builds a predictive model using ML algorithms for predicting the individual risk of HTN in Ethiopia. Thus, the objective of this study was to develop an efficient ensemble based explainable ML framework for predicting patients with the risk of HTN in Ethiopia.

**Fig 1:**
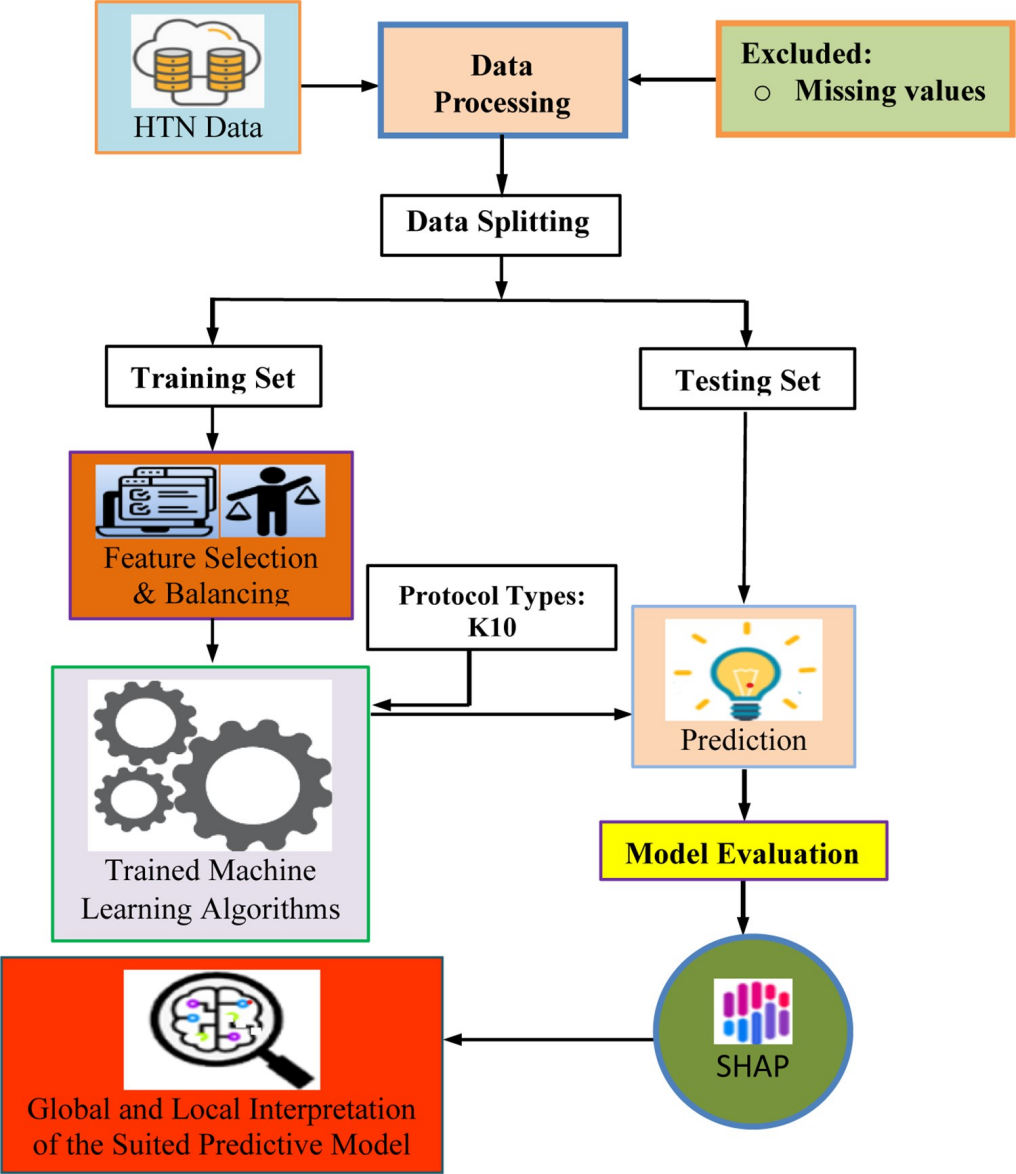
Workflow of the proposed ML-based methodology for predicting risk of HTN.

Furthermore, we employed under-sampling and adaptive syntactic (ADASYN) class balancing strategy to enhance the confidence score of the developed prediction models. For model interpretation, we identified the key risk factors of HTN and direction of the relationship between the risk factors and HTN using SHapley Additive exPlanations (SHAP), which is a post hoc model interpretation technique viz. theoretically based on the Shapley value. The overall pipeline of the explainable machine learning based framework is displayed in Fig 1.

The layout of this paper is presented as follows: Materials and methods included data source, statistical analysis, feature selection, machine learning algorithms, performance evaluation criteria, and model interpretability. The results are presented in section 3 and discussed in section 4. Finally, conclusion is represented in section 5.

## Materials and methods

### Data source

The community-based cross-sectional data used in this investigation were collected in 2017 by the Hawassa city administration and made available to the public by Paulose et al. [39]. The data were collected through multistage random sampling and comprised a total of 633 respondents, ranging in age from 31 to 90, and residing in the city for at least six months. The sample size was determined by using the formula of sample size determination method, which considered the design effect of 1.5, the 95% confidence interval, the 5% margin of error, the 80% power, the proportion of 50% (to maximize sample size), and the 10% non-response rate [39]. Different levels of explanatory variables were included as individual risk factors of HTN and categorized the quantitative variables based on the previous sittings [18–20, 39]. A brief explanation of the included risk factors has been presented in Table 1. In this study, a patient with HTN is determined based on WHO cutoff (≥140/90 mmHg and/or diastolic pressure ≥90 mmHg and/or being on medication of HTN at the time of data collection) [40]. Finally, a total of 612 respondents were incorporated in this study after eliminating all the missing values.

**Table 1:** Name, description, and categorization of the selected factors.

SN	Name	Description	Categorization
1	Residence	Permanent Residence	Urban, Semi-urban
2	Sex	Sex of the respondents	Male, Female
3	Age	Age of the respondents	Continuous variable (year)
4	MS	Marital status	Single, Married, Divorced, Widowed
5	Religion	Religion status	Protestant, Orthodox, Catholic, Muslim, Others
6	Ethnicity	Ethnicity	Sidama, Walayita, Kembata, Guraga, Amahra, Oromo, Hadiya
7	Education	Education level	Cannot read and write, Read and write only, Primary, Secondary, Diploma and above
8	Occupation	Occupation status	Employee, Daily-labor, Merchant, Housewife, Retired, Others
9	FM	Family member	1–3, 4–6, 7 or 7+
10	Income	Average monthly income	Continuous variable (Birr)
11	PA	Physical activity	Yes, No
12	Walking	Walking at least 10 minutes	Yes, No
13	Diabetes	Having diabetes mellitus	Yes, No
14	Height	Height of the respondents	Continuous variable (cm)
15	Weight	Weight of the respondents	Continuous variable (kg)
16	BMI	Body mass status	Underweight, Normal, Overweight, Obese
17	Smoking	Smoking status	Yes, No
18	Drinking	Drinking alcohol	Yes, No
19	Kchat	Ever chew kchat	Yes, No
20	Fruit	Eat fruit at least per week	Yes, No
21	Vegetable	Eat fruit at least per week	Yes, No
22	Fat	Having fat	Yes, No
23	Salt	Eating habit salt	Yes, No
24	Transport	Mode of transport	On foot/pedal bicycle, Engine
25	HD	History of diabetes	Yes, No
26	Wealth	Wealth status	Poorest, Very poor, Poor, Less poor, Least poor
27	HHTN	History of hypertension	Yes, No

### Statistical analysis

The baseline and demographic characteristics of the patients were presented in percentage (%) for categorical and mean ± SD (standard deviation) for continuous data. Pearson χ^2^-test was employed to determine the association between categorical risk factors and HTN, whereas for continuous risk factors, independent sample t-test was used to examine the mean difference between the HTN groups (HTN vs. non-HTN) for normally distributed data. Two-sided test was performed and a p-value of <0.05 was considered statistically significant for all the tests. Data analysis was performed by SPSS (version-27.0) and R (version-4.2.2).

### Feature selection

Feature selection (FS), or risk factor identification is also known as variable selection, or subset selection in statistics and ML. The identification of risk factors is a method for selecting the relevant features by removing the irrelevant or redundant features from the dataset. In this study, Boruta-based feature selection method (FSM) was adopted to identify the relevant features. Boruta is a wrapper-based feature selection method that employs the random forest classifier algorithm. This method has a wider range of applications and performs better than others as it is unbiased and steady [41].

### Machine learning algorithms

This study used three different types of supervised ML algorithms for predicting patients with the risk of HTN (Table 2).

**Table 2:** Different machine learning algorithms with types.

Types	Algorithms
Classical	Logistic regression (LR)
Non-linear	Artificial neural network (ANN)
Ensemble	Random forest (RF) and extreme gradient boosting (XGB)

### Logistic regression

Logistic regression (LR) is a most popular supervised ML-based algorithm that leverages the idea of probability. Logistic regression (LR) is a most popular supervised ML algorithm mainly used for classification task [42]. The LR model employs the logistic function to estimate the probability of the response variable (HTN and non-HTN) in terms of one or more input features. The logistic function can be represented as follows

$$\mathrm{logit}(p_{j})=\log_{e}\left(\frac{p_{j}}{1-p_{j}}\right)=\beta_{0}+\beta_{1}x_{1j}+\beta_{2}x_{2j}+\ldots ..+\beta_{k}x_{kj}+\epsilon_{j},j=1,2,\ldots ,n$$ (1)

where, *p*_*j*_ denote the probability of HTN and (1−*p*_*j*_) denote the probability of non-HTN for j^th^ individual; *X*_*kj*_ is the k^th^ input feature of the j^th^ individual and β_*k*_ is the k^th^ regression coefficients.

The above Eq (1) can be expressed as

$$p=\frac{exp(\beta_{0}+\beta_{1}x_{1j}+\beta_{2}x_{2j}+\ldots ..+\beta_{k}x_{kj})}{1+{\exp(\beta }_{0}+\beta_{1}x_{1j}+\beta_{2}x_{2j}+\ldots ..+\beta_{k}x_{kj})}$$ (2)

and odds as

$$\frac{p}{1-p}=exp(\beta_{0}+\beta_{1}x_{1j}+\beta_{2}x_{2j}+\ldots ..+\beta_{k}x_{kj})$$ (3)

If $\frac{p}{1-p}>1$, then we classify as HTN, while $\frac{p}{1-p}<1$, then we classify as non-HTN.

### Artificial neural network

Artificial neural network (ANN) is a non-linear modeling algorithm that is inspired by the structure and function of human brain. It consists of interconnected processing nodes that are organized by three different types of layers: input, hidden, and output. The input layer is connected to hidden layer with updated weight, and hidden layer is connected to the output. In this method, *X* = *x*_1_,…,*x*_*k*_ are used as the input vector in back propagation (BP) algorithm for learning as well as mapping the relationship between input features and outcome variable. The BP algorithm propagates the error between the input risk factors and outcome variable by adjusting weights of hidden layers via backward direction with non-linear sigmoid activation function [43]. The sigmoid activation function is defined as

$$Sigmoid(x)=\frac{1}{1+e^{-x}}.$$ (4)

This procedure is repeated iteratively until no change iteration values or not getting the minimum error.

### Random forest

Random forest is a popular machine learning algorithm that developed by Leo Breiman and widely used in classification and regression problems [44]. It is based on the concept of ensemble learning algorithm that trains multiple decision tree on random subsets of the data to solve the problem. The RF-based model is constructed by using the following steps:

Step1: The given training data set (X_ij_, i = 1, 2… k, j = 1, 2… n), select randomly risk factors from training dataset by using bootstrap sampling procedure.
Step 2: Built a decision tree (DT) for creating new subset.
Step3: Repeat Step1 and Step2, until construct many trees and consist of a forest.
Steps 4: Consider the prediction result from each created DT and select final prediction with the help of majority voting.

### Extreme gradient boosting

Extreme gradient boosting (XGB) is an efficient ensemble-based machine learning algorithm that uses decision trees and gradient boosting algorithm. It is highly adaptable and working in most classification problem, especially HTN disease prediction [45]. Boosting is a learning algorithm, which attempts to create a strong classifier based on weak learners or classifiers. The weak and strong classification models mention to the correlation of predicted and actual class. By adding classifiers on top of each other iteratively, the next classifier can modify the errors of the earlier one. This procedure is repeated until the training data set accurately predicts the membership class label of the target variable.

### Data partition and balancing

We randomly divided the whole dataset into two sets as 70% training set [HTN: 91 (21.2%), non-HTN: 338 (78.8)] and 30% testing set HTN: 39 (21.3%), non-HTN: 144 (78.7)] using stratified sampling procedure [46]. Membership class label of the data was imbalance i.e., skewed class distribution of observations. Imbalance class problem of a data provided a biased result for the majority class of the response variable in classification task [47, 48]. To deal this problem, several data balancing strategy are widely applicable. Among them, under-sampling and Adaptive synthetic (ADASYN) balancing strategy were executed in the training set to balance the data. ADASYN is the newly generalized version of synthetic minority oversampling technique (SMOTE) and generates new sample for the minority class using a weighted distribution [49].

### Cross validation and tune hyperparameters

The mentioned above four ML algorithms (LR, ANN, RF, and XGB) have other parameters, called hyperparameters. Hyperparameters are those parameters that the user explicitly defines before the learning process to improve the model performance. The grid search method with repeated10-fold (K10) cross-validation protocol was used to tune the hyperparameter values in the training set. The training dataset is divided into a 9:1 ratio as a training subset and a verification set to perform the K10 protocol. The caret package (version 6.0-93) in R was used to generate the optimal hyperparameter values for four models, which are displayed in Table 3.

**Table 3:** The value of hyperparameter for ML-based models.

Models	Hyperparameter
LR	c = (0.001, 0.01, 0.1, 1, 10,100,1000)
ANN	size = (1, 2, 3, 4, 5, 6, 7,8, 9, 10); decay = (0, 0.1, 0.01, 0.001, 0.001)
RF	mtry = (1, 2, 3, 4, 5, 6, 7, 8, 9, 10, 11, 12, 13, 14, 15, 16, 17, 18, 19, 20)
XGB	nrounds = (100, 500); max_depth = (10, 15, 20, 25, 30); colsample_bytree = seq (0.5, 0.9, length.out = 5); eta = 0.1; gamma = 0; min_child_weight = 1; subsample = 1.

### Performance evaluation criteria

The performance of selected four ML models was evaluated by five popular evaluation criteria: accuracy, precision, recall, F-score, and area under the curve (AUC). The values of performance evaluation criteria were calculated from the confusion matrix by four measures (Table 4):

True positive (*t*_*p*_): model predicted the disease group as HTN where actual group was HTN,
False positive (*f*_*p*_): model predicted the disease group as HTN where actual group was non-HTN,
False negative (*f*_*n*_): model predicted the control group non-HTN where actual class was HTN,
True negative (*t*_*n*_): model predicted the control group non-HTN where actual group was non-HTN.

**Table 4:** Confusion matrix.

		Actual class	
	Total cases/population = HTN + non-HTN	HTN	non-HTN
**Predicted class**	HTN	True positive (*t*_*p*_)	False positive (*f*_*p*_)
	non-HTN	False negative (*f*_*n*_)	True negative (*t*_*n*_)

#### Accuracy

It is used to assess the overall accuracy for the models. It is defined as the ratio of the sum of true cases (*t*_*p*_ and *t*_*n*_) against total number of cases. Accuracy is defined mathematically as

$$\mathrm{Accuracy}(\%)=(\frac{t_{p}+t_{n}}{t_{p}+t_{n}+f_{p}+f_{n}})\times 100$$ (5)

#### Precision

It is the ratio of t_p_ cases against the predicted positive (DR) cases. It is also called positive predictive value and used to assess the reliability for predicting the model as positive. Precision is defined mathematically as

$$\mathrm{Precision}(\%)=(\frac{t_{p}}{t_{p}+f_{p}})\times 100$$ (6)

#### Recall

It is the ratio of *t*_*p*_ cases against the actual positive cases (DRs). Model with high recall indicates low *f*_*n*_. It’s also called sensitivity or true positive rate (TPR). Recall is defined mathematically as

$$\mathrm{Recall}(\%)=(\frac{t_{p}}{t_{p}+f_{n}})\times 100$$ (7)

#### F1-score

It is a harmonic mean of precision and recall. F-score is defined mathematically as

$$F1‐\mathrm{score}(\%)=(\frac{2t_{p}}{{2t}_{p}+f_{p}+f_{n}})\times 100$$ (8)

### Area under the curve

The AUC is defined as an integral of the receiver operating characteristic (ROC) function over the given range and used to assess the quality of the built predictive model. The mathematical formula of AUC is as follows

$$AUC=\int_{x=0}^{1}TPR({FPR}^{-1}\;(x))dx$$ (9)

A ROC curve is a plot of TPR or *sensitivity* on the y axis against false positive rate (FPR) or *1-specificity* on the x axis for different cutoff values. The ROC curve is broadly used in medical diagnosis as another single-number measure for evaluating the predictive validity of ML-based model [50]. ROCs generate an AUC value from 0 to 1.

### Model interpretability

Shapley additive explanations (SHAP) is an interpretability visualization approach, which is constructed based on Shapley values. This method was introduced by Lundberg and Lee (2017), and widely used to explain the local and global importance using SHAP value by computing the contribution of each risk factor in the ML-based prediction model [51]. The explanation value of SHAP was initially established from coalitional game theory, where each predictor is used as an individual player in a game or coalition. SHAP values framework offers a fair solution for each player in a model outcome, and provides a series of desirable properties/axioms, including consistency, efficiency, dummy, and additively [52]. The efficiency property of SHAP method provided better reliable results compared to another methods, for example local interpretable model-agnostic explanations [53]. Risk factors contribute to the model’s outcome or prediction with different magnitude and sign, which is accounted for by Shapley values. Accordingly, Shapley values represent estimates of feature importance magnitude of the contribution and its direction (sign). Risk factors with positive SHAP value contribute to predict patent with HTN in the model, whereas risk factors with negative SHAP value contribute to predicting patients with control in the model. Particularly, the importance of each risk factor, say *k*^*th*^ risk factor, is measured by the Shapley value defined by the following formula

$$\emptyset_{k}(v)=\frac{1}{M!}\sum_{S\subseteq M\setminus \{k\}}\left|S|!(M-|S|-1)![v(S\cup \{k\})-v(S)\right]$$ (10)

where, *S* denotes the subset of risk factors, that does not include the risk factor for which we are calculating the value of ∅_*k*_(*v*); *S*∪{*k*} is the subset of risk factors, that includes in *S* and the *k*^*th*^ risk factor; *v*(*S*) corresponds to the outcome of the ML-based model that explain using the risk factors of *S*; *S*⊆*M*\{*k*} represents all sets of *S* that are subsets of the full set of *M* risk factors, excluding the *k*^*th*^ risk factor.

## Results

### Baseline characteristics

This study enrolled 612 participants (HTN: 130, 21.2% and non-HTN: 482, 78.8%) with 27 HTN-related predictor variables (Table 5). About 53.4% respondents were male and more than half of the respondents living in urban areas. The average age of the participants was 47.56.20±13.40 years, height 165.20±8.87 cm, and weight 66.589±8.769 kg. Obese respondents showed higher prevalence rate of HTN than normal (50.0% vs. 13.4%). Patients having diabetes (47.5% vs. 30.0%) and smoking (50.4% vs. 23.8%) were more prevalent to HTN. The prevalence of HTN was greater among the respondents who had family history of diabetes (41.8% vs. 11.2%) and HTN (60.3% vs. 21.9%). The result of association showed that residence, sex, age, occupation, income, PA, walking, diabetes, height, weight, BMI, smoking, drinking, vegetable, fat, salt, transport, HD, wealth, HHTN were significantly associated with HTN (P-value<0.005).

**Table 5:** Baseline characteristic of the respondents.

Risk factors	Total	HTN	Non-HTN	P-value
Overall, n (%)	612	130 (21.2)	482 (78.8)	
Residence, n (%)				
Urban	408(66.7)	100(24.5)	308(75.5)	0.006
Semi-urban	204(33.3)	30(14.7)	174(85.3)	
Sex, n (%)				
Male	327(53.4)	85(26.0)	242(74.0)	0.002
Female	285(46.6)	45(15.8)	240(84.2)	
Age, mean (SD)	47.56(13.40)	54.66 (14.04)	45.64(12.56)	<0.001
MS, n%				
Single	29(4.74)	4(13.8)	25(86.2)	0.795
Married	500(81.70)	108(21.6)	392(78.4)	
Divorced	24(3.92)	5(20.8)	19(79.2)	
Widowed	59(9.64)	13(22.0)	46(78.0)	
Religion, n (%)				
Protestant	337(55.07)	62(18.4)	275(81.6)	0.147
Orthodox	181(29.58)	50(27.6)	131(72.4)	
Catholic	38(6.21)	6(15.8)	32(84.2)	
Muslim	50(8.17)	11(22.0)	39(78.0)	
Other	6(0.98)	1(16.7)	5(83.3)	
Ethnicity, n (%)				
Sidama	222(36.27)	43(19.4)	179(80.6)	0.285
Walayita	146(23.86)	26(17.8)	120(82.2)	
Kembata	105(17.16)	24(22.9)	81(77.1)	
Guraga	58(9.48)	14(24.1)	44(75.9)	
Amahra	44(7.19)	15(34.1)	29(65.9)	
Oromo	27(4.41)	7(25.9)	20(74.1)	
Hadiya	10(1.63)	1(10.0)	9(90.0)	
Education, n (%)				
Cannot read and write	139(22.71)	33(23.7)	106(76.3)	0.595
Read and write only	98(16.01)	16(16.3)	82(83.7)	
Primary education (1–8)	84(13.73)	16(19.0)	68(81.0)	
Secondary education (9–12)	108(17.65)	22(20.4)	86(79.6)	
Diploma and above	183(29.90)	43(23.5)	140(76.5)	
Occupation, n (%)				
Employee	199(32.52)	32(16.1)	167(83.9)	0.033
Daily-laborer	53(8.66)	9(17.0)	44(83.0)	
Merchant	165(26.96)	43(26.1)	122(73.9)	
Housewife	118(18.28)	22(18.6)	96(81.4)	
Retired	59(9.64)	20(33.9)	39(66.1)	
Others	18(2.94)	4(22.2)	14(77.8)	
FM, n (%)				
1–3	138(22.55)	22(15.9)	116(84.1)	0.062
4–6	281(45.92)	57(20.3)	224(79.7)	
7 or 7+	193(31.54)	51(26.4)	142(73.6)	
Income, mean (SD)	3169.69 (1999.468)	3664.23 (2503.960)	3036.31(1820.149)	0.001
PA, n (%)				
Yes	371(60.6)	41(11.1)	330(88.9)	<0.001
No	241(39.4)	89(36.9)	152(63.1)	
Walking, n (%)				
Yes	471(77.0)	63(13.4)	408(86.6)	<0.001
No	141(23.0)	67(47.5)	74(52.5)	
Diabetes, n (%)				
Yes	561(91.67)	98(17.5)	463(82.5)	<0.001
No	51(8.33)	32(62.7)	19(37.3)	
Height, mean (SD)	165.20(8.871)	168.98(8.062)	164.18(8.811)	<0.001
Weight, mean (SD)	66.59(8.769)	73.07(9.462)	64.84(7.698)	<0.001
BMI, n (%)				
Underweight	10(1.63)	2(10.0)	8(80.0)	<0.001
Normal	366((59.80)	49(13.4)	317(86.6)	
Overweight	212(34.64)	67(31.6)	145(68.4)	
Obese	24(3.92)	12(50.0)	12(50.0)	
Smoking, n (%)				
Yes	74(12.1)	29(29.2)	45(60.8)	<0.001
No	538(87.9)	101(18.8)	437(81.7)	
Drinking, yes, n (%)				
Yes	141(23.0)	51(36.2)	90(63.8)	<0.001
No	471(77.0)	79(16.8)	392(83.2)	
Kchat, yes, n (%)				
Yes	526(85.9)	107(20.3)	419(79.7)	0.200
No	86(14.1)	23(26.7)	63(73.3)	
Fruit, no, n (%)				
Yes	429(70.1)	89(20.7)	340(79.3)	0.667
No	183(29.9)	41(22.4)	142(77.6)	
Vegetable, no, n (%)				0.011
Yes	524(85.6)	102(19.5)	422(80.6)	
No	88(14.4)	28(31.8)	60(68.2)	
Fat, yes, n (%)				<0.001
Yes	188(69.3)	61(32.4)	127(67.6)	
No	424(30.7)	61(32.4)	127(67.6)	
Salt, yes, n (%)				<0.001
Yes	81(13.2)	31(38.3)	50(61.7)	
No	531(86.8)	99(18.6)	432(81.4)	
Transport, n (%)				
On foot/pedal bicycle	313(51.1)	51(16.3)	262(83.7)	0.003
Engine	299(48.9)	79(26.4)	220(73.6)	
HD, n (%)				
Yes	51(8.3)	32(62.7)	19(37.3)	<0.001
No	561(91.7)	98(17.5)	463(82.5)	
Wealth, n (%)				
Poorest	170(27.78)	21(12.4)	149(87.6)	0.011
Very poor	103(16.83)	24(23.3)	79(76.7)	
Poor	117(19.12)	29(24.8)	88(75.2)	
Less poor	161(26.31)	37(23.0)	124(77.0)	
Least poor	61(9.97)	19(31.1)	42(68.9)	
HHTN, n (%)				
Yes	84(13.7)	45(53.6)	39(46.4)	<0.001
No	528(86.3)	85(16.1)	443(83.9)	

### Risk factors selection using Boruta

**Fig 2:**
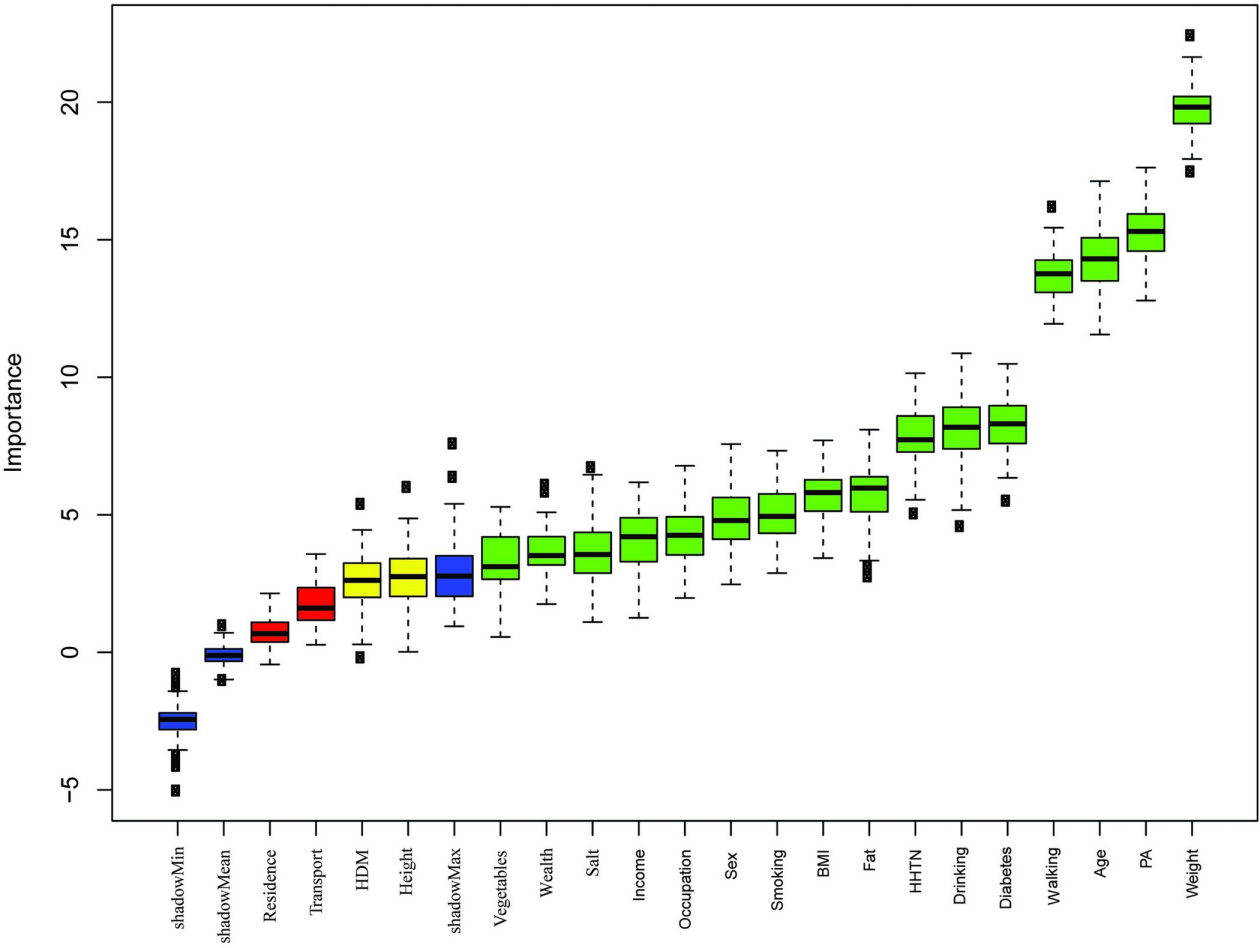
Risk factors selection using Boruta based feature selection method.

The result of Boruta based feature selection method is presented in Fig 2. The method showed that age, occupation, PA, walking, diabetes, height, weight, BMI, smoking, drinking, vegetable, fat, transport, HD, wealth, and HHTN were the important risk factors of HTN. The selected risk factors were included to construct the ML-based model for prediction of HTN status (HTN or non-HTN).

### Performance comparisons of ML-based models

The performance of four ML-based models with under-sampling and ADASYN shown in Table 6 and S1 Fig. It is to be noticed that XGB model with ADASYN balancing method achieved the highest predictive discrimination ability with the accuracy of 88.81% (95% CI: 85.44–91.63), precision of 89.62, recall of 97.04, F1-score of 93.18, and AUC of 0.894 (95% CI: 0.827–0.961) compared to others.

**Table 6:** Performance of four models with two class balancing methods.

Balancing methods	Models	Accuracy	Precision	Recall	F1-score	AUC
Under-sampling	LR	83.61(77.43–88.66)	87.08	96.44	91.52	0.848(0.789–0.929)
	ANN	84.15(78.04–89.12)	86.02	96.04	91.20	0.843(0,788–0.928)
	RF	85.81(79.88–90.50)	87.42	96.13	92.02	0.863(0.797–0.931)
	XGB	86.89(81.12–91.41)	88.23	96.35	92.51	0.871 (0.794–0.937)
**ADASYN**	LR	86.43(81.74–91.86)	88.05	97.22	92.41	0.863(0.809–0.941)
	ANN	85.25(79.26–90.05)	87.80	96.67	92.02	0.874(0.816–0.943)
	RF	87.88(84.41–90.81)	88.65	97.04	92.66	0.880(0.806–0.895)
	**XGB**	**88.81(85.44–91.63)**	**89.62**	**97.04**	**93.18**	**0.894(0.827–0.961)**

**Fig 3:**
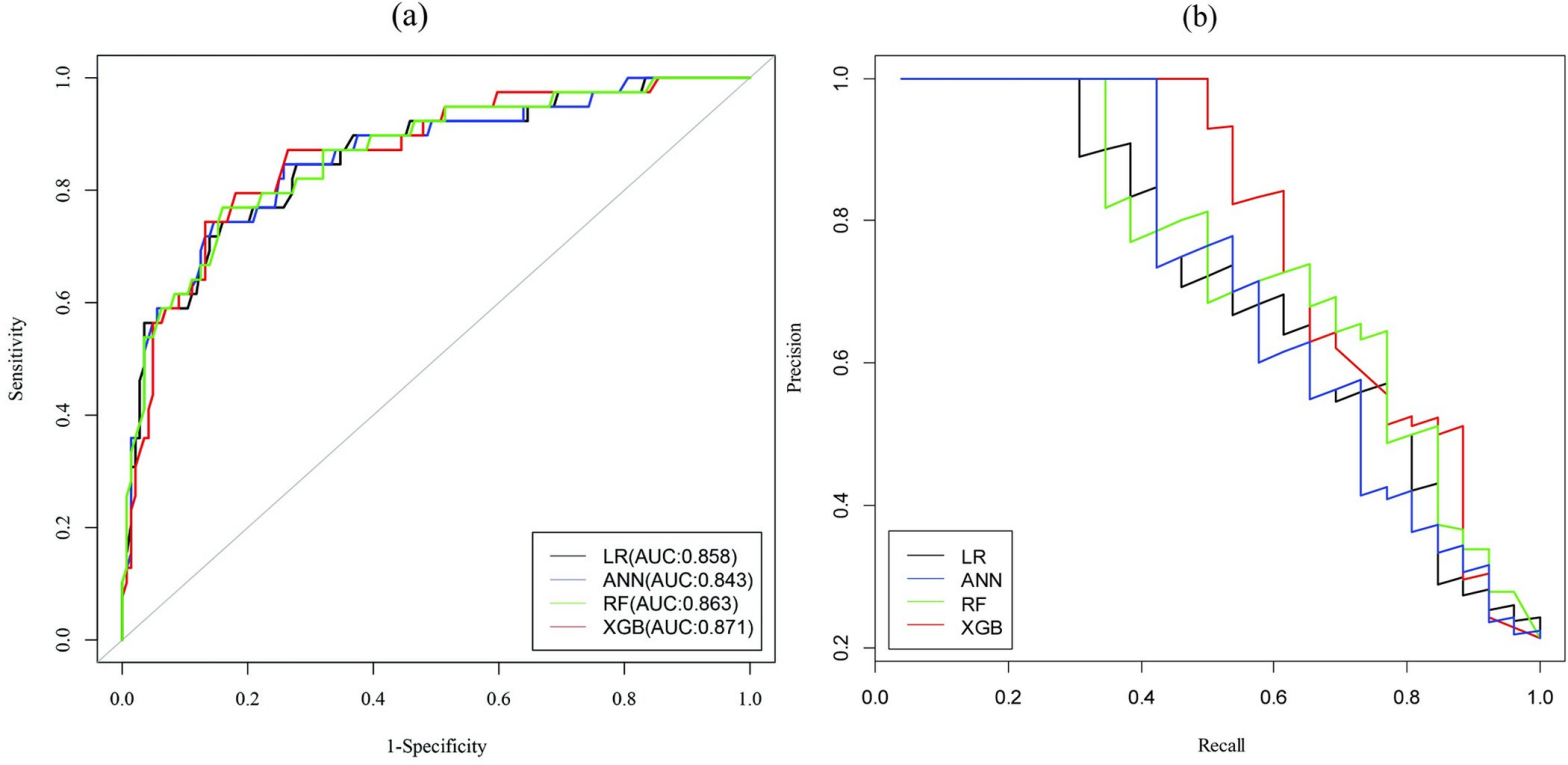
(a) ROC curves and (b) Precision vs. recall curves of four predictive models.

The corresponding ROC curves and precision recall curves of four predictive models with ADASYN displayed in Fig 3. The ROC curves and precision recall curves also indicated that the XGB model reached significantly better than other models as LR, ANN, and RF. Therefore, in comparison to other models, our results showed that the XGB-based model with ADASYN performed well.

### Interpretable risk factors of hypertension

**Fig 4:**
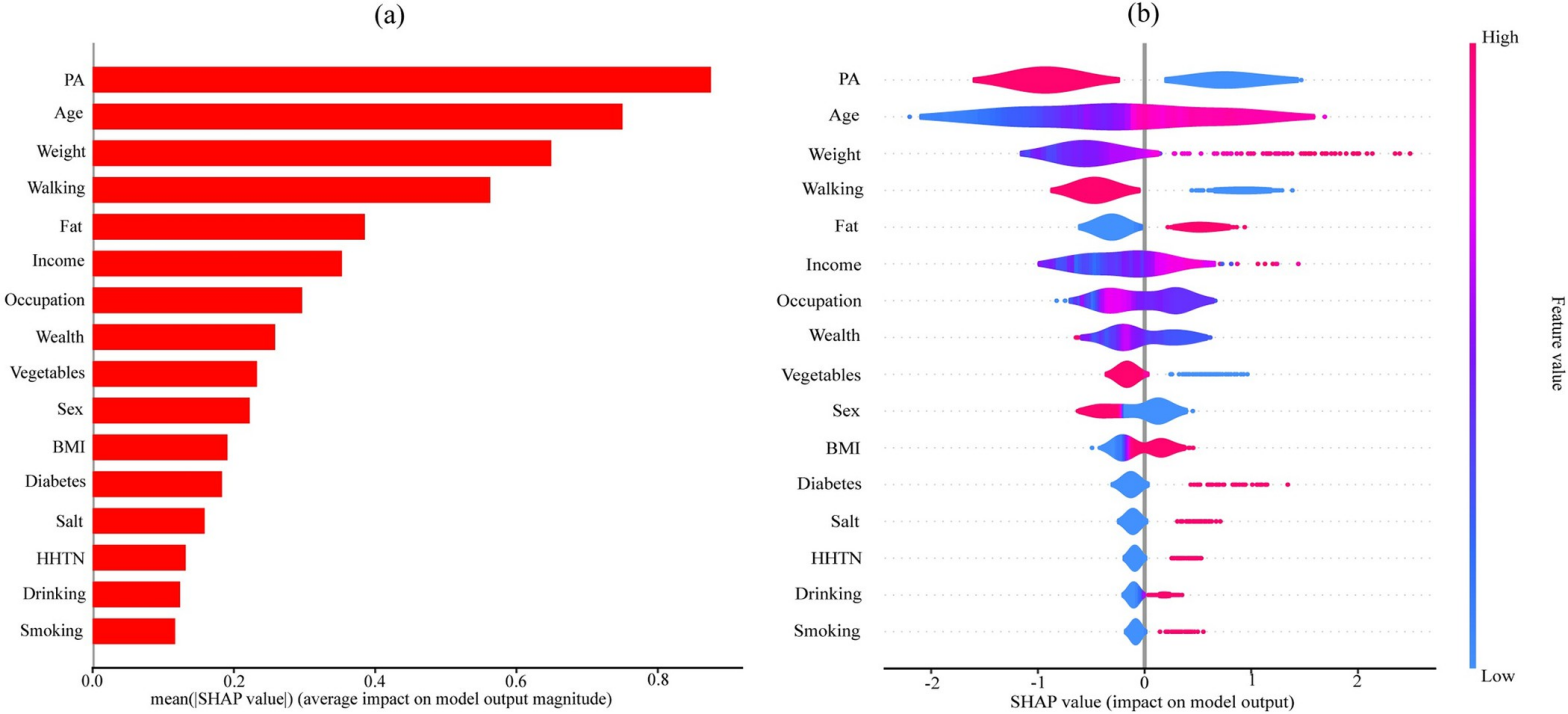
Importance of risk factors based on SHAP values. (A) Mean absolute SHAP values, to explain global risk factor importance, (B) Local explanation summary, to reveal the direction of the relationship between a risk factor and game outcome.

SHAP analysis was executed to determine the interpretable predictive risk factor of HTN for the suited prediction model (XGB) based on the SHAP values. Fig 4(A) explains the global importance of each risk factor of XGB-based model. The importance plots only show the global influence of each feature on the prediction. However, the global importance plot does not indicate which risk factors affect positively (HTN) or negatively (non-HTN) on the prediction. For that reason, summary plots are executed, which provide a global macro-level explanation of how the input risk factors contribute to the prediction. Fig 4(B) represents the summary plot indicating the importance, impact, original value, and correlation of the risk factors to high risk of HTN. Particularly, the effect [positive (HTN) vs. negative (non-HTN)] is shown on the *x*-axis. The color signifies the value of a specific risk factor, wherein red indicates a high value and blue indicate a low value. However, XGB-based model showed that age, weight, fat, income, BMI, diabetes, salt, HHTN, drinking, and smoking were the high interpretable risk factors on the predication of HTN.

## Discussion

In this study, we investigate several ML-based algorithms to propose an explainable framework for predicting the risk of HTN in Ethiopia. We trained up four ML algorithms (ANN, SVM, RF, and XGB) to predict HTN, using 16 risk factors obtained from Boruta feature selection method. The performance of the developed models compared by accuracy, precision, recall, F1-score, and ROC curve with AUC value on testing set. Based on performance measurements, we proposed XGB model as the most appropriate candidate classifier for predicting HTN.

Several studies were conducted using ML framework to predict the risk of HTN. A comparison of the present study with the existing studies is presented in Table 7. Chowdhury et al. [54] proposed a system on 18,322 respondents with 24 candidate risk factors in Canada. Before constructing the models, they applied five top FSM for selecting the significant risk factors and adopted five ML algorithms LASSO, Elastic Net, random survival forest (RSF), and gradient boosting, with the conventional Cox proportional hazard model for predicting HTN. They measure the performance of the models by C-index for each model. Pratiwi OA [35] applied four ML algorithms such as DT, RF, GB, and LR for predicting individual risk of HTN in Indonesia. He developed the model by K10 protocol based on training set and prediction performance of these models was measure on testing set in terms of accuracy, precision, recall, F1-score, and AUC. He indicated LR is the best performer marginally compared to others with AUC (0.829). Oanh and Tung [55] suggested a ML based model to predict patient with the risk of HTN in Vietnam. The model was developed by Naïve Bayes (NB), multilayer perceptron (MLP), decision tree (DT), k-nearest neighbors (kNN), SVM, and ensemble algorithms: bagging (RF), boosting and voting based on training set. The performance of the models was assessed by testing set in terms of F1-score, precision, and recall. Islam et al. [38] conducted a study on three countries such as Bangladesh, Nepal, and India. They included 818603 respondents with seven risk factors and performed GT, RF, GBM, XGB, LR, LDA algorithms for predicting HTN patients. They focused that XGB achieved the best performance score than others. Chai et al. [56] used Malaysian data with 2461 respondents and 11 covariates to develop a system for diagnosing HTN patients by 3 different types of algorithms, including neural network (MLP), classical model (LR, DT, NB, k-NN), and ensemble model (RF, SVM, GB, XGB, LightGBM, CatBoost, AdaBoost, and LogitBoost). Before building the model, they adopted correlation-based FSM to select a set of leading features and utilized SMOTE technique to balance membership class label of the data. They evaluate the predictive ability of the models by sensitivity, specificity, accuracy, precision, F1-score, misclassification rate, and AUC on testing set and found that LightGBM based model acquired the best accuracy with 74.39%. Islam et al. [57] used nationally representative HTN data in Bangladesh. The data consisted of 6965 subjects with 13 risk factors. They determine the prominent risk factors of HTN by two popular FSM such as LASSO and SVMRFE in Bangladesh. They utilized then K10 protocol to construct model using four ML algorithms on training set and measured the performance of the models on testing set using accuracy, precision, recall, F1- score and AUC. Overall experimental sittings demonstrated that gradient boosting model attained the best score of AUC (0.669). Zheng et al. [58] explored a system for predicting HTN patients using several ML techniques in USA. No feature selection method had used to select the prominent features of HTN before constructing ML-based system. They found that ANN model reached the maximum performance score. Alkaabi et al. [59] utilized HTN data in Qatar. The dataset comprised of 987 respondents with 12 risk factors. They adopted 3 ML-based algorithms including DT, RF, and LR. Overall experimental results anticipated that RF model provided better generalization predictive ability than others.

**Table 7:** Comparative performance of the proposed study with the existing studies.

Authors	Year	Country	Data size	# of risk factors	Algorithms	AUC	SHAP
Chowdhury et al. [54]	2023	Canadian	18,322	24	penalized regression Ridge, Lasso, Elastic Net (EN), random survival forest (RSF), and GB	NA	No
Pratiwi [35]	2022	Indonesia	30,320	11	DT, RF, GB, **LR**	0.829	No
Oanh & Tung [55]	2022	Vietnam	2509	10	Naïve Bayes, MLP, Decision Tree, kNN and SVM; and **RF**, boosting and voting	NA	No
Islam et al. [38]	2022	Bangladesh, Nepal, India	818603	7	GT, RF, GBM**, XGB,** LR, LDA	NA	No
Chai et al. [56]	2022	Malaysia	2461	11	LR, DT, RF, SVM, NB, kNN, MLP, GBM, XGB, **LightGBM**, CatBoost, AdaBoost, LogitBoost	0.686	No
Islam et al. [57]	2021	Bangladesh	6965	13	ANN, DT, RF, **GB**	0.669	No
Zheng et al. [58]	2021	USA	500	17	LR, SMV, DTR, GPR, **ANN**	NA	No
AlKaabi et al. [59]	2020	Qatar	987	12	DT, **RF**, LR	NA	No
**Proposed**		Ethiopia	612	27	LR, ANN, RF, **XGB**	0.894	Yes

Thus, the comparative results suggested that our proposed XGB framework can predict HTN with higher AUC (Table 7). Moreover, SHAP analysis with the proposed method revealed that age, weight, fat, income, diabetes, BMI, height, salt, smoking, and HHTN were the associated risk factors for developing HTN. Local explanation summary plot showed that age is the 1^st^ leading risk factor of HTN in Ethiopia. A study conducted by Belay et al., [2022] in Ethiopia found that a patient with age>60 years was two times more likely to have HTN than those with age 18–40 years [11]. This result also supported by several systematic review and meta-analysis studies [60, 61]. The vascular system of our body changes in arteries, particularly with large artery stiffness caused by older age. Weight and fat are the 2^nd^ and 3^rd^ leading drivers of HTN. This finding supports the conclusions of earlier investigations [62]. Excess body weight increases visceral and retroperitoneal fat, which can contribute to the development of HTN. Household income is linked to the risk of HTN, which was in line with the prior investigations [63]. Due to a number of reasons, including the ongoing nutritional transition, rising trends in sedentary lifestyle, and other modifiable risk factors, people from low-income families may have a greater burden from the disease [64]. BMI is another gradient of HTN which is corroborated with the earlier studies [65]. BMI might be a cause of HTN and other cardiovascular disease by stimulating the renin-aldosterone system and endothelial dysfunction [66]. Diabetes is another important marker of HTN. The two medical conditions diabetes and HTN may cause each other and share common risk factors. HHTN is another important covariate of HTN. This result is also coincided with the previous studies conducted in Ethiopia and other countries [67]. This might be as family member share same genetic factors, behaviors, mostly similar lifestyle, and environments related factor that could influence the risk of HTN disease. Additionally, other risk factors such as salt, drinking alcohol, and smoking were found to be an important contributing risk factors of HTN, which is similar with other studies in literature [68, 69]. Although this work has many strengths, it also has some limitations, such as the sample only included permanent the residents of the city administration who had lived in the area for more than six months and were older than 30. Additionally, it did not measure the amount of alcohol, cigarettes, fruits, vegetables, fats, and salts that were consumed in measurable units.

## Conclusions

In this study, we adopted four different machine learning algorithms to build the most appropriate predictive model for classification of HTN. Overall experimental results anticipated that, among four models, the XGB model is the most appropriate model for predicting patient with the risk of HTN. The SHAP analysis revealed that age, weight, fat, income, BMI, diabetes, salt, HHTN, drinking, and smoking are the high contributing risk factors for developing HTN. Therefore, the proposed integrating system can be conveniently utilized as a useful tool in clinical sittings to accurately identify the patients with the risk of HTN at an early stage. With the help of this information, a doctor can make decisions that will reduce healthcare costs and time while also enabling individualized interventions and targeted treatment to minimize the burden of HTN in Ethiopia.

## Supporting information

S1 Fig ROC curve of four models with two class balancing methods, (a) under-sampling and (b) ADASYN.
(DOCX)
