# Supplementary figures and images for: Predicting the risk of hypertension using machine learning algorithms: A cross sectional study in Ethiopia
Source: PLoS One. 2023 Aug 24;18(8):e0289613. doi: 10.1371/journal.pone.0289613 (PMC10449142; doi:10.1371/journal.pone.0289613)

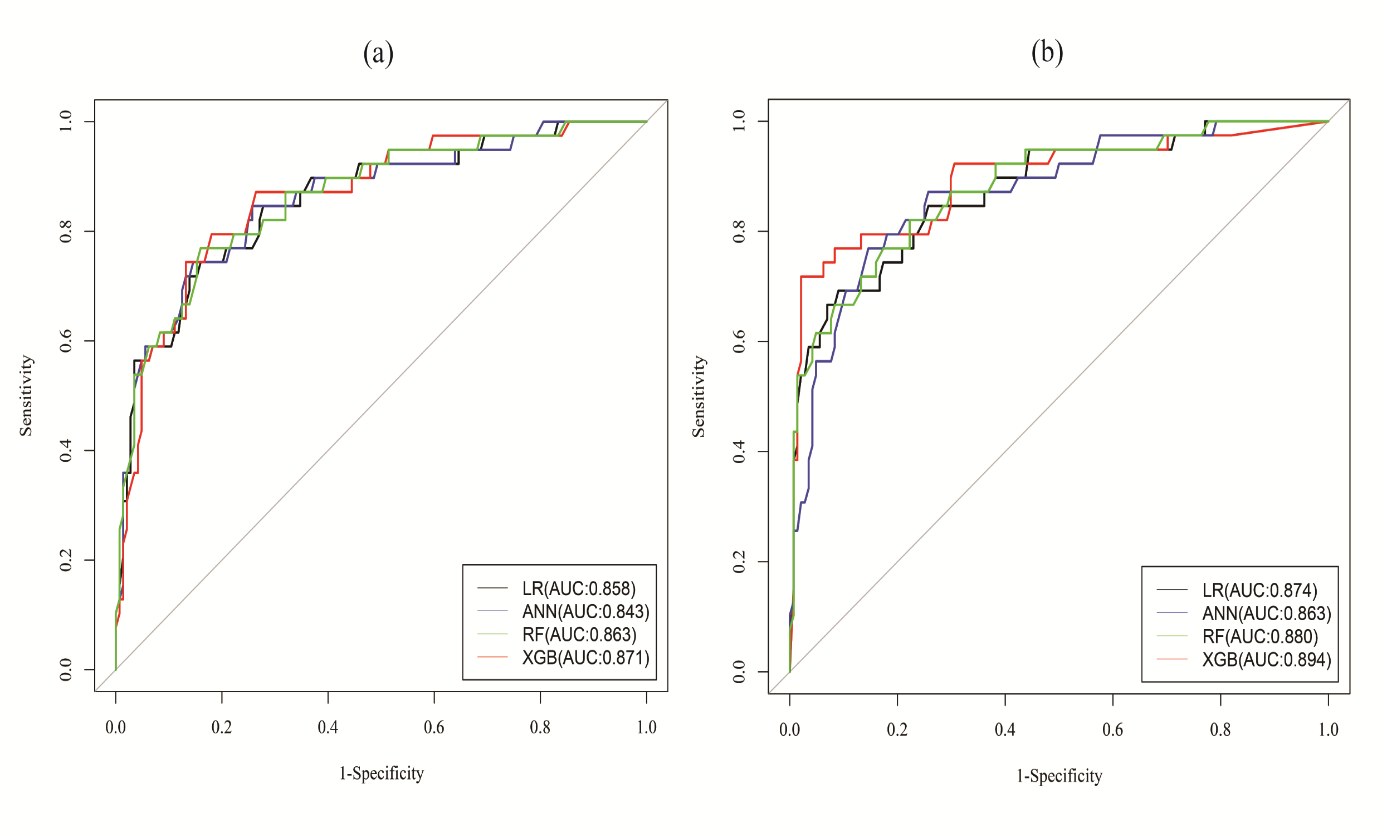


**S1 Fig. ROC curve of four models with two class balancing methods, (a) under-sampling and (b) ADASYN.**

Supplement: S1 Fig — ROC curve of four models with two class balancing methods, (a) under-sampling and (b) ADASYN. (DOCX) [file pone.0289613.s001.docx]
